# Supplementary material for: Scaffold-free 3D-cell co-culture model system for the study of metastatic cancer in the brain TME
Source: PLoS One. 2026 May 11;21(5):e0349061. doi: 10.1371/journal.pone.0349061 (PMC13160345; doi:10.1371/journal.pone.0349061)

SUPPLEMENTARY INFORMATION

S1 Figure. Z-stack imaging of 3D-endothelial/cancer cell constructs

Scaffold-free 3D-Cell Co-Culture Model System for the Study of Metastatic Cancer in the Brain TME

Pratistha Sarkar,[1] Shreya Ahuja,[1#] and Iulia M. Lazar*[1,2,3,4]

[1]Department of Biological Sciences, 1981 Kraft Drive, Blacksburg, VA 24061, USA

[2]Fralin Life Sciences Institute/Virginia Tech, [3]Carilion School of Medicine/Virginia Tech, and

[4]Division of Systems Biology/Academy of Integrated Science/Virginia Tech, USA

***Correspondence:** Iulia M. Lazar

E-mail: malazar@vt.edu

(A) Z-stack imaging of 3D-endothelial/cancer cell constructs (HBEC-5i/SK-OV-3-GFP)

- HBEC-5i cells were seeded in T25 flasks.
- After 15 days of HBEC-5i monoculture, SK-OV-3/GFP cells were added to the HBEC-5i cells.
- SK-OV-3/GFP cells migrate toward and cluster on the high-density areas of HBEC-5i constructs.
- Z-stack images were acquired at 8 days after adding SK-OV-3/GFP cells to the HBEC-5i monocultures (4X objective, 25 µm interval acquisition).
- Cell culture media: DMEM HG/ 10% FBS/ 0.5% Pen-Strep.
- Best focus for most top SK-OV-3/GFP cells was achieved at ~200 µm.

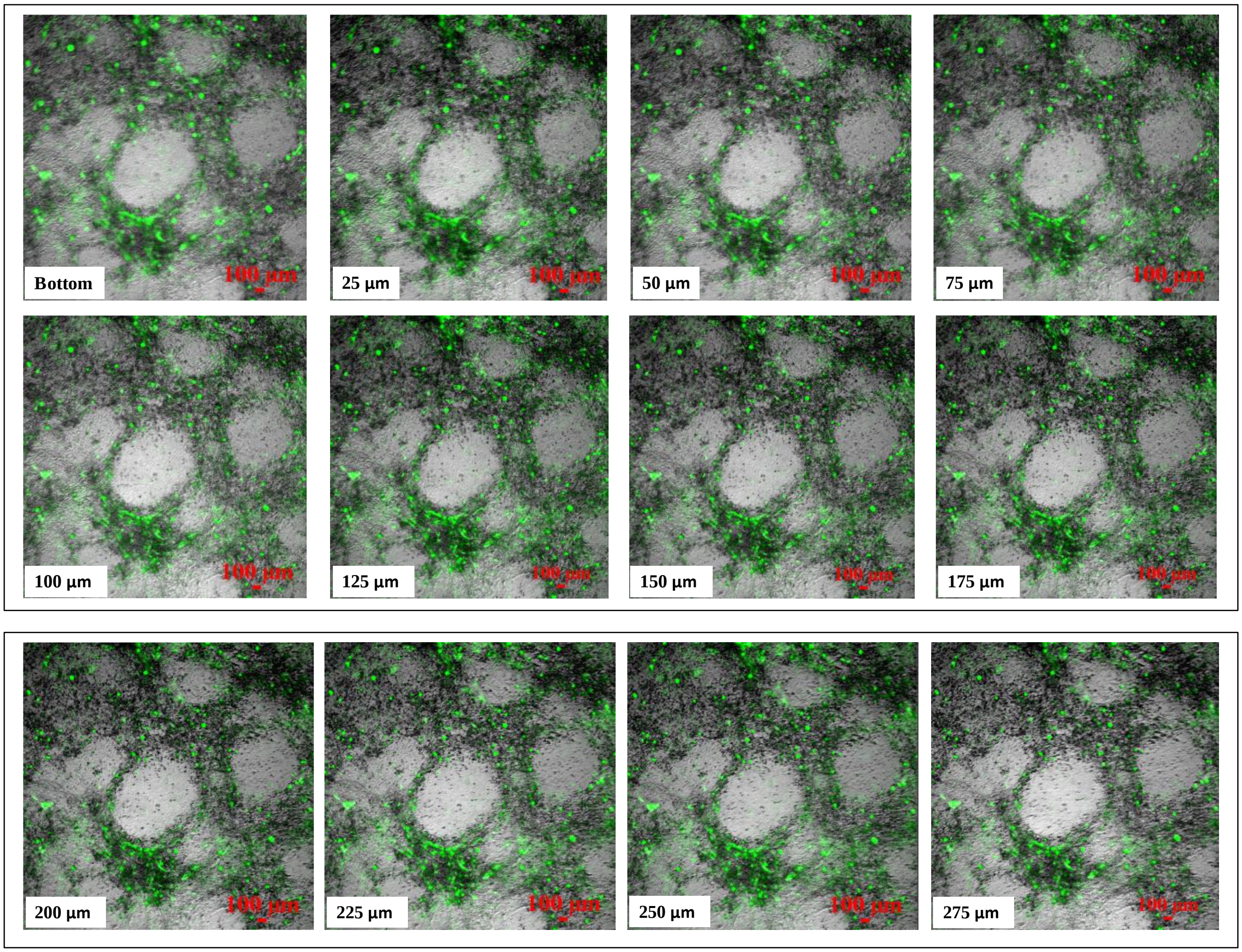

(B) Z-stack imaging of 3D-endothelial/cancer cell constructs (HBEC-5i/SK-OV-3-GFP)

- HBEC-5i cells were seeded in T25 flasks.
- After 15 days of HBEC-5i monoculture (higher confluence than in A), SK-OV-3/GFP cells were added to the HBEC-5i cells.
- SK-OV-3/GFP cells migrate toward and cluster on the high-density areas of HBEC-5i constructs.
- Z-stack images were acquired at 2 days after adding SK-OV-3/GFP cells to the HBEC-5i monocultures (4X objective; 25 µm interval acquisition/shown at 50 µm intervals).
- Cell culture media: DMEM HG/ 10% FBS/ 0.5% Pen-Strep.
- Best focus for most top SK-OV-3/GFP cells was achieved at ~400-500 µm; we estimate that the localization of the bottom layer of cells was subject to a spatial uncertainty of ~100 µm, induced by limitations in focusing on the highly confluent and densely packed underlying endothelial cells.

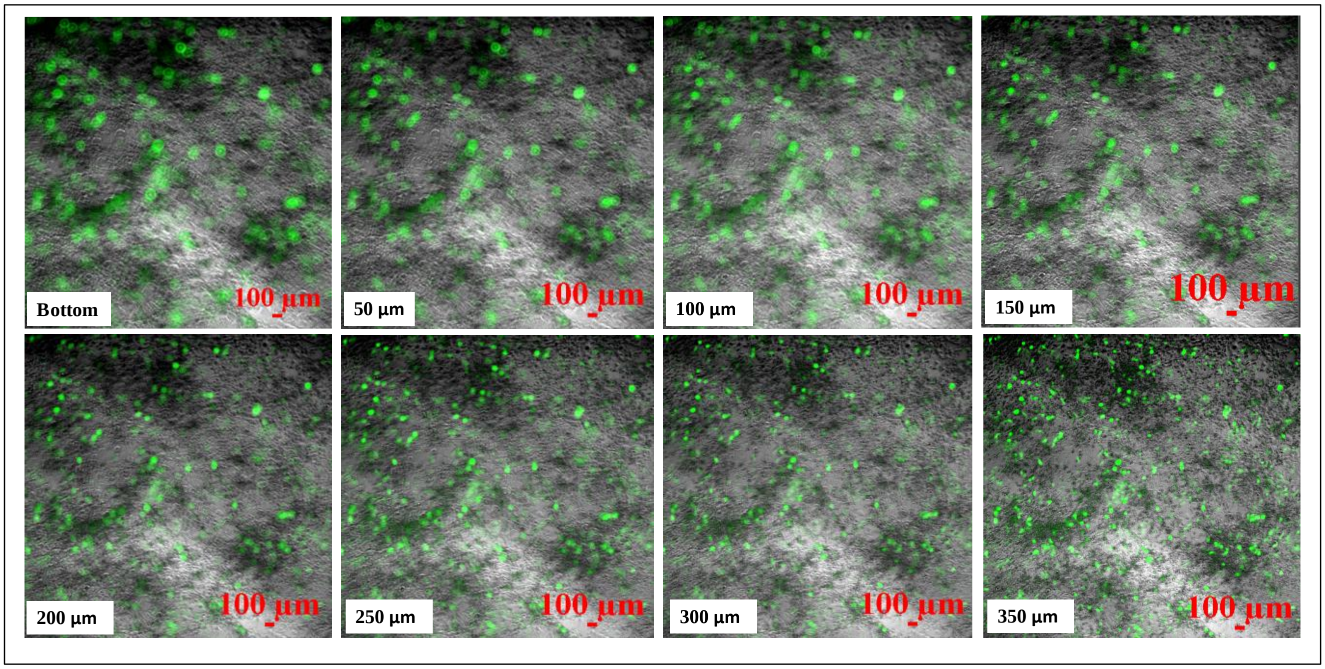

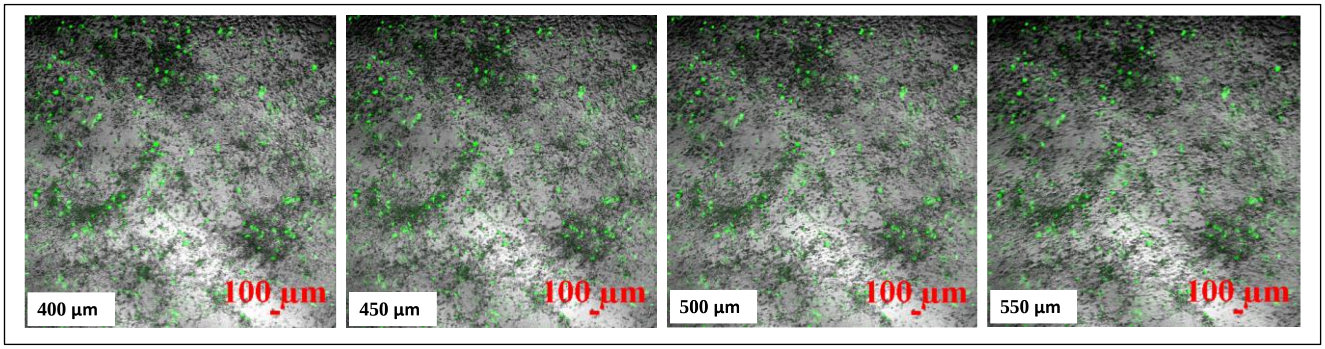

Supplement: S1 Fig — (PDF) [file pone.0349061.s001.pdf]
