# Supplementary material for: Scaffold-free 3D-cell co-culture model system for the study of metastatic cancer in the brain TME
Source: PLoS One. 2026 May 11;21(5):e0349061. doi: 10.1371/journal.pone.0349061 (PMC13160345; doi:10.1371/journal.pone.0349061)

SUPPLEMENTARY INFORMATION

S2 Figure. Network topology measurements of 3D endothelial/cancer cell constructs

Scaffold-free 3D-Cell Co-Culture Model System for the Study of Metastatic Cancer in the Brain TME

Pratistha Sarkar,[1] Shreya Ahuja,[1#] and Iulia M. Lazar*[1,2,3,4]

[1]Department of Biological Sciences, 1981 Kraft Drive, Blacksburg, VA 24061, USA

[2]Fralin Life Sciences Institute/Virginia Tech, [3]Carilion School of Medicine/Virginia Tech, and

[4]Division of Systems Biology/Academy of Integrated Science/Virginia Tech, USA

***Correspondence:** Iulia M. Lazar

E-mail: malazar@vt.edu

Summary

Branch length

Set #	n	Range (Min-Max)	Mean (SD)	Median (QR1-QR3)
1	40	250-705	449 (123)	422 (355-524)
2	35	305-921	528 (164)	471 (416-635)
3	30	324-1750	823 (322)	792 (599-1052)
4	18	231-1594	767 (401)	689 (423-1031)

Branch width

Set #	n	Range (Min-Max)	Mean (SD)	Median (QR1-QR3)
1	40	29-94	57 (14)	59 (48-64)
2	35	40-95	70 (17)	70 (58-83)
3	30	50-194	111 (38)	96 (87-134)
4	18	45-206	79 (40)	64 (54-95)

(A) Co-culture networks prepared from premixed HBEC-5i and GFP-transfected SK-OV-3 cells (1:1).

- Image acquired after 23 days of co-culture (Figure 2 in the manuscript).
- Branch length dimensions: median (442 µm), mean (449 µm)/standard deviation (123 µm).
- Branch width dimension: median (59 µm), mean (57 µm)/standard deviation (14 µm).

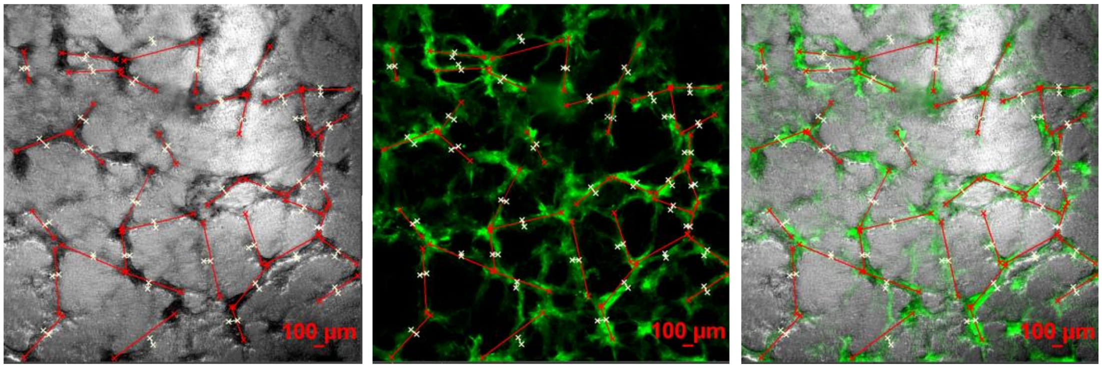

(B) Co-culture networks prepared from premixed HBEC-5i and GFP-transfected SK-OV-3 cells (1:1).

- Image acquired after 28 days of co-culture, with PI stain (Figure 7 in the manuscript).
- Branch length dimensions: median (471 µm), mean (528 µm)/standard deviation (164 µm).
- Branch width dimension: median (70 µm), mean (70 µm)/standard deviation (17 µm).

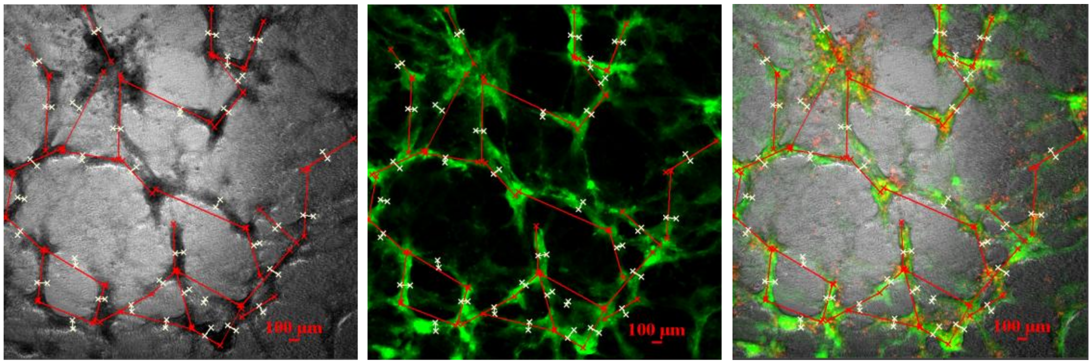

(C) Co-cultures prepared from pre-formed HBEC-5i networked structures to which GFP-transfected SK-OV-3 cells were added.

- Image acquired after 20 days of co-culture (Figure 4 in the manuscript).
- Branch length dimensions: median (792 µm), mean (823 µm)/standard deviation (322 µm).
- Branch width dimension: median (96 µm), mean (111 µm)/standard deviation (38 µm).

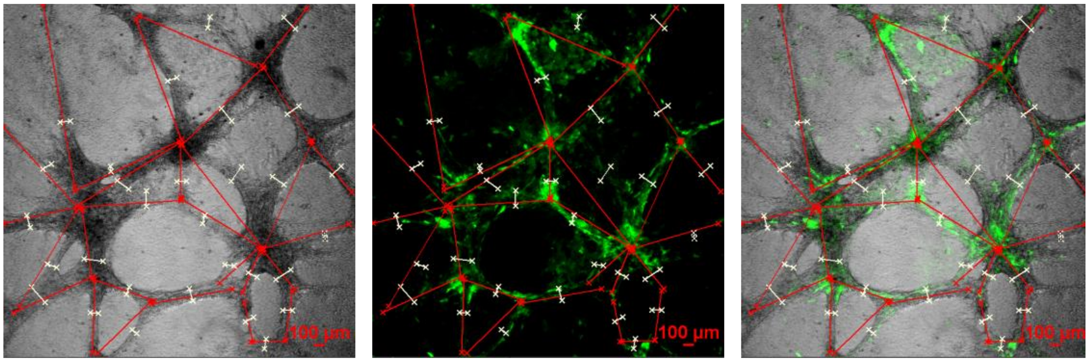

(D) Co-cultures prepared from premixed HBEC-5i and GFP-transfected SK-OV-3 cells (9:1).

- Image acquired after 23 days of co-culture.
- Branch length dimensions: median (689 µm), mean (767 µm)/standard deviation (401 µm).
- Branch width dimension: median (64 µm), mean (79 µm)/standard deviation (40 µm).

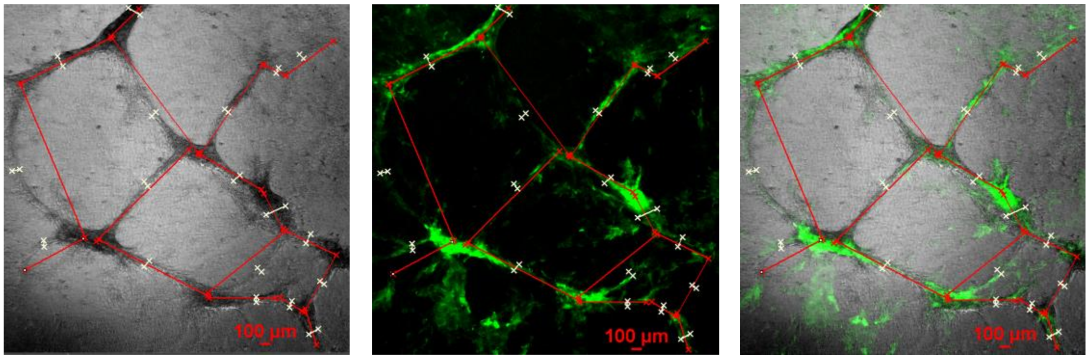

Supplement: S2 Fig — (PDF) [file pone.0349061.s002.pdf]
