# Supplementary material for: Scaffold-free 3D-cell co-culture model system for the study of metastatic cancer in the brain TME
Source: PLoS One. 2026 May 11;21(5):e0349061. doi: 10.1371/journal.pone.0349061 (PMC13160345; doi:10.1371/journal.pone.0349061)
Supplement: S3 Fig — (PDF) [file pone.0349061.s003.pdf]

SUPPLEMENTARY INFORMATION

S3 Figure. Images of HBEC-5i cultures and 3D constructs exposed to different concentrations of FITC-labeled dextran and incubation times

Scaffold-free 3D-Cell Co-Culture Model System for the Study of Metastatic Cancer in the Brain TME

Pratistha Sarkar,[1] Shreya Ahuja,[1#] and Iulia M. Lazar*[1,2,3,4]

[1]Department of Biological Sciences, 1981 Kraft Drive, Blacksburg, VA 24061, USA

[2]Fralin Life Sciences Institute/Virginia Tech, [3]Carilion School of Medicine/Virginia Tech, and

[4]Division of Systems Biology/Academy of Integrated Science/Virginia Tech, USA

***Correspondence:** Iulia M. Lazar

E-mail: malazar@vt.edu

HBEC-5i monoculture
Cell culture medium: DMEM-HG / FBS 10 % / Pen-Strep 0.5 %
FITC-dextran stain: 50 µg/mL
Incubation time: 1 day
FITC-dextran staining is observed in FITC-excited areas of low density HBEC-5i cells
The opaque 3D constructs reflect an accumulation of HBEC-5i cells rather than the presence of tubular structures

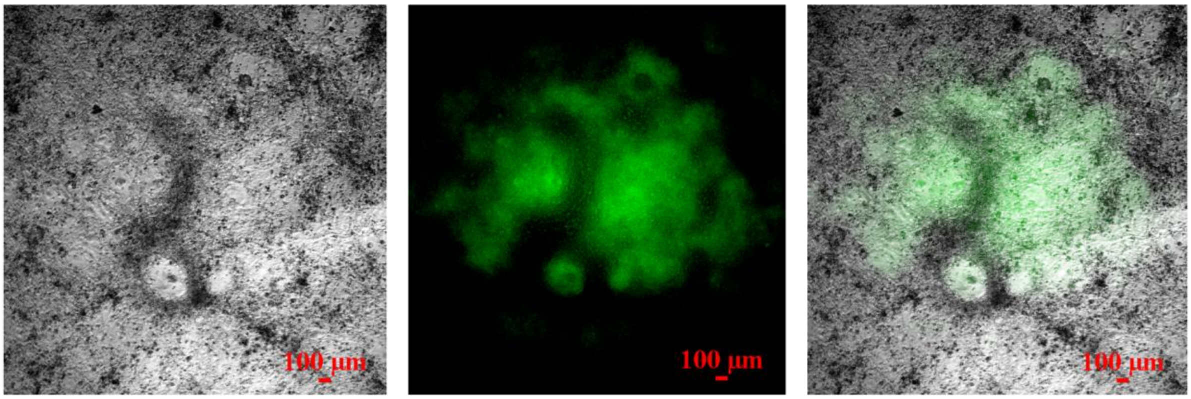

Transmitted light **FITC filter** **Merged**

HBEC-5i monoculture
Cell culture medium: DMEM-HG / FBS 10 % / Pen-Strep 0.5 %
FITC-dextran stain: 200 µg/mL
Incubation time: 30 min
FITC-dextran staining is observed in FITC-excited areas of low density HBEC-5i cells
The opaque 3D constructs reflect an accumulation of HBEC-5i cells rather than the presence of tubular structures

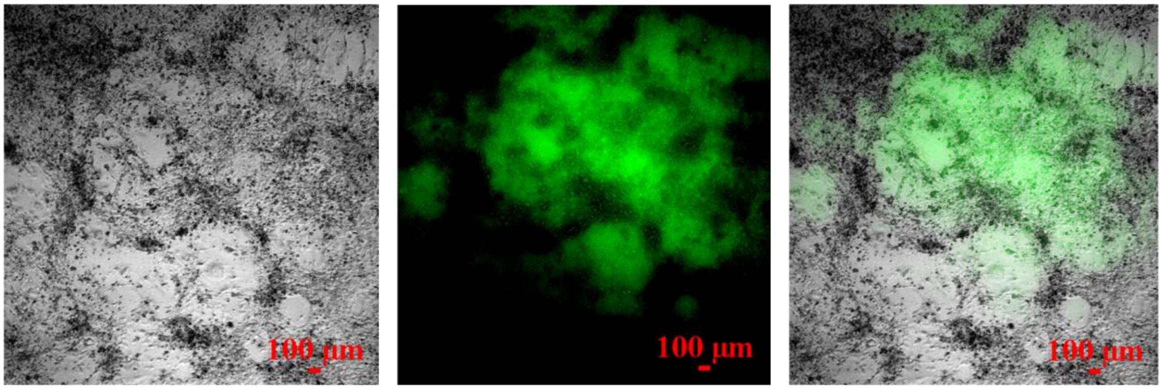

Transmitted light **FITC filter** **Merged**

HBEC-5i monoculture
Cell culture medium: DMEM-HG / FBS 10 % / Pen-Strep 0.5 %
FITC-dextran stain: 500 µg/mL
Incubation time: 30 min
FITC-dextran staining is observed in FITC-excited areas of low density HBEC-5i cells
The opaque 3D constructs reflect an accumulation of HBEC-5i cells rather than the presence of tubular structures

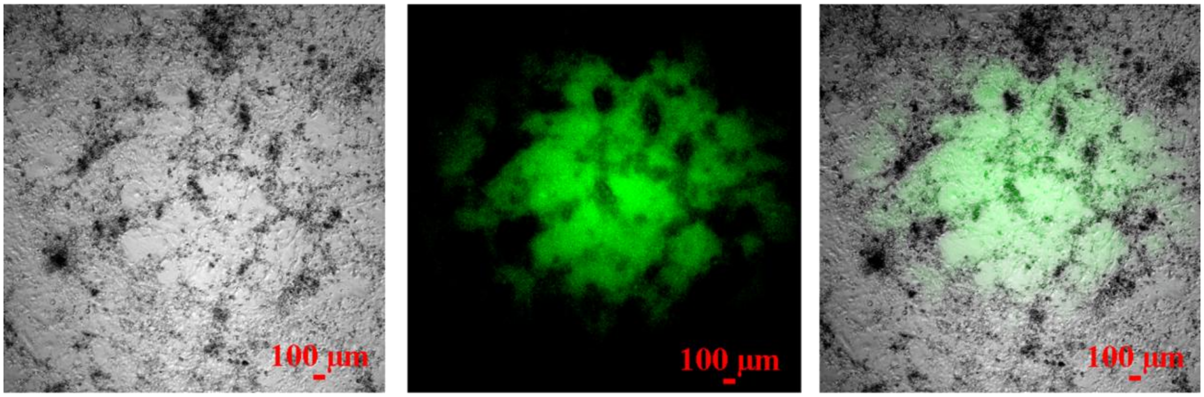

Transmitted light | **FITC filter** | **Merged**

HBEC-5i monoculture
Cell culture medium: DMEM-HG / FBS 10 % / Pen-Strep 0.5 %
FITC-dextran stain: 500 µg/mL
Incubation time: 1 day
FITC-dextran staining is observed in FITC-excited areas of low density HBEC-5i cells
The opaque 3D constructs reflect an accumulation of HBEC-5i cells rather than the presence of tubular structures

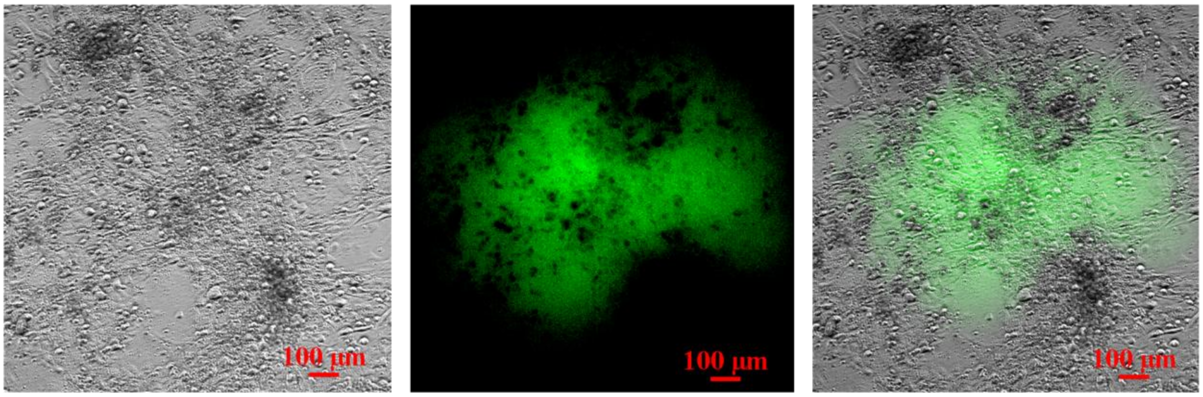

Transmitted light | **FITC filter** | **Merged**
